# The drug war must end: The right to life, liberty and security of the person during the COVID-19 pandemic for people who use drugs

**DOI:** 10.1186/s12954-021-00474-8

**Published:** 2021-02-17

**Authors:** VANDU, WAHRS, BCAPOM, Russ Maynard, Ehsan Jozaghi

**Affiliations:** 1 The Vancouver Area Network of Drug Users (VANDU), 380 E Hastings St, Vancouver, BC V6A 1P4 Canada; 2 The Western Aboriginal Harm Reduction Society (WAHRS), 380 E Hastings St, Vancouver, BC V6A 1P4 Canada; 3 The British Columbia Association People on Opiate Maintenance (BCAPOM), 380 E Hastings St, Vancouver, BC V6A 1P4 Canada; 4 PHS Community Services Society, 9 East Hastings St., Vancouver, BC V6A 1M9 Canada; 5 UBC Faculty of Dentistry|Nobel Biocare Oral Health Centre, 2151 Wesbrook Mall, Vancouver, BC V6T 1Z3 Canada

**Keywords:** People who use drugs, The war on drugs, Human rights, Opioid epidemic, Overdose, Decriminalization, Canada, Health, Marginalization, Criminalization, COVID-19, Safe supply, Fentanyl

## Abstract

Since the start of the opioid epidemic in 2016, the Downtown Eastside community of Vancouver, Canada, has lost many pioneering leaders, activists and visionaries to the war on drugs. The Vancouver Area Network of Drug Users (VANDU), the Western Aboriginal Harm Reduction Society (WAHRS), and the British Columbia Association People on Opiate Maintenance (BCAPOM) are truly concerned about the increasing overdose deaths that have continued since 2016 and have been exacerbated by the novel coronavirus (SARS-COVID-19) despite many unique and timely harm reduction announcements by the British Columbia (B.C.) government. Some of these unique interventions in B.C., although in many cases only mere announcements with limited scope, are based on the philosophy of safe supply to illegal street drugs. Despite all the efforts during the pandemic, overdose deaths have spiked by over 100% compared to the previous year. Therefore, we urge the Canadian federal government, specifically the Honorable Patty Hajdu, the federal Minister of Health, to decriminalize simple possession immediately by granting exemption under *the Controlled Drugs and Substances Act*. The Canadian federal government has a moral obligation under Sect. 7 of *the Canadian Charter of Rights and Freedoms* to protect the basic human rights of marginalized Canadians.

## Background

Since 2016, the start of the synthetic opioid epidemic, we at the Vancouver Area Network of Drug Users (VANDU), the longest running harm reduction and peer-run drug user advocacy group in North America, have lost countless leaders in our community in the war on drugs [1]. Vancouver’s Downtown Eastside community has produced the founding of the Western Aboriginal Harm Reduction Society (WAHRS), the British Columbia Association People on Opiate Maintenance (BCAPOM), and the SALOME-NAOMI Association of Patients. These are powerful examples of leadership in the civil rights of drug users and would not have been possible without the tireless activism of our comrades and friends who lost their lives too soon [2, 3].

## Drug overdose deaths during COVID-19 pandemic

We at VANDU, WAHRS, and BCAPOM are alarmed at the B.C. and Federal government’s inaction on the increasing mortality of our members and of drug users throughout Canada. Overdose cases in B.C. linked to powerful synthetic opioids have spiked dramatically during pandemic conditions; this despite several novel announcements by the provincial government to provide an alternative, pharmaceutical, supply for drug users asked to self-isolate due to the COVID-19 pandemic [4]. These strategies [5]—permitting physicians to prescribe other substituting therapies (such as hydromorphone and dextroamphetamine)—*have not been successful in reducing the overdose mortalities in the Province of B.C.*, as the latest figures show an average of over 5 deaths per day linked to drug overdoses [4–7]. In reality, these efforts at creating alternative prescribing have yet to be implemented anywhere near consistently or on a scale that reflects the scale of the epidemic; particularly in rural or other parts of the province [8, 9]. The opioid crisis has deepened during the COVID-19 pandemic. Recent data from the B.C. Coroners Service reported a 116% increase in mortalities linked to toxic illegal drugs in October 2020 compared to October 2019 [4]. Government action to end the drug war is needed more than ever. In this 21st year of the twenty-first century we desperately need a national drug policy based on science and the lived experience of users.

## Next step: full decriminalization

We therefore support the unanimous vote by Vancouver City Council in November to fully decriminalize small amounts of illegal drugs for personal use, and urge the federal government to approve their request, without delay or modification [10]. This vote follows the call by the Canadian Association of Chiefs of Police and the Toronto Board of Health, who also urged Ottawa to decriminalize simple possession in June, 2020 [11]. We believe that Honorable Patty Hajdu, the federal Minister of Health, not only has the power to grant an exemption under *the Controlled Drugs and Substances Act* to allow decriminalization across Canada, but that she is morally obliged to do so immediately to protect the rights to life, liberty and security of person—Sect. 7 of *The Canadian Charter of Rights and Freedoms*—so that people who use drugs are given a chance to live.

## Conclusion

VANDU, WAHRS, and BCAPOM have been advocating for the full decriminalization of small possession for personal use for decades. While we in the drug users’ liberation movement feel vindicated in seeing the government’s long-overdue first steps towards decriminalization, we mourn for the lives of those who were needlessly lost in the drug war. We mourn, but we continue to organize. Decriminalization is just the beginning of our fight to live and flourish with dignity.
